# Eco-friendly activated carbon derived from pomegranate peel for amoxicillin removal: batch adsorption, kinetic modeling, and thermodynamics

**DOI:** 10.1038/s41598-026-51191-w

**Published:** 2026-05-23

**Authors:** Nazan YILMAZ

**Affiliations:** Department of Chemical Engineering, Faculty of Engineering, Afyon Kocatepe University, Afyonkarahisar, Turkey

**Keywords:** Wastewater, Amoxicillin, Activated carbon, Adsorption, Removal, Chemistry, Environmental sciences

## Abstract

Activated carbon derived from waste pomegranate peels was investigated for the removal of amoxicillin (AMX) from aqueous solutions. The prepared adsorbent exhibited a high BET surface area (1307 m²/g) and a well-developed micro–mesoporous structure. Under optimal conditions (pH 2, 0.05 g, 25 °C, 50 mg/L), a maximum removal efficiency of 97% was achieved, while 87% removal was maintained at near-neutral pH (pH 6). Increasing the initial concentration reduced removal efficiency due to adsorption site saturation, whereas increasing temperature decreased adsorption, confirming the exothermic nature of the process. Kinetic studies showed that the pseudo-second-order model provided the best fit, indicating that surface-controlled interactions govern the adsorption rate. Equilibrium data were better described by the Freundlich model, suggesting heterogeneous adsorption behavior, although the Langmuir model also indicated a high monolayer adsorption capacity (qₘₐₓ = 100 mg g⁻¹). Thermodynamic parameters (ΔG° = −6.13 to − 5.16 kj/mol, ΔH° = −15.59 kj/mol, ΔS° = −32.29 J/mol K) confirmed that the adsorption process is spontaneous and exothermic. The relatively low ΔH° value indicates that adsorption is predominantly governed by physisorption mechanisms. Overall, the results demonstrate that this low-cost and sustainable adsorbent is a promising alternative for efficient antibiotic removal from water.

## Introduction

The widespread use of antibiotics in human and veterinary medicine has led to their continuous release into aquatic environments, where they persist and contribute to the formation of antibiotic-resistant bacterial strains. Intensive use of antibiotics in both sectors results in the excretion of a substantial fraction of unchanged drugs, which enter wastewater systems^1^. Among these compounds, amoxicillin (AMX), a broad-spectrum β-lactam antibiotic, is frequently detected in domestic wastewater, hospital effluents, and surface waters. Conventional wastewater treatment plants are not specifically designed to remove pharmaceutical residues; as a result, significant amounts of AMX can pass through treatment processes unchanged and enter natural water bodies^2^. This situation raises substantial environmental and public-health concerns, emphasizing the need for effective and economical removal methods^3^.

Adsorption has emerged as one of the most promising techniques for the removal of antibiotics from water due to its operational simplicity, high efficiency, regeneration capability, and relatively low cost. Activated carbon (AC), in particular, is widely used owing to its high surface area and well-developed pore structure^4^. Consequently, the adsorption of amoxicillin using various commercial activated carbons, bio-based carbons, mineral-modified adsorbents, and engineered materials has been extensively investigated. For example, studies have reported the use of olive biomass–derived activated carbon for AMX removal (surface area up to ~ 1742 m²/g with fast kinetics)^5^. Other bio-based adsorbents such as chitosan-derived porous carbon have also shown high adsorption capacity for AMX^6^. Moreover, reviews on biomass/biochar-based adsorbents highlight a wide range of materials—including agricultural wastes—utilized for antibiotic removal, underscoring the versatility and efficiency of activated carbon in this field^5^. Activated carbons from non-traditional sources, like argan waste, have likewise been demonstrated to remove AMX effectively, with high adsorption capacity and favorable kinetics^7^. Biomass-derived activated carbons have garnered increasing attention due to their renewability, low production cost, and reduced environmental footprint. Among these biomass sources, pomegranate peel represents an abundant agro-industrial waste rich in lignocellulosic content and carbon-precursor compounds, making it a suitable candidate for activated carbon production. Indeed, a study reported the preparation of activated carbon from pomegranate peel coated with zero-valent iron nanoparticles and its application for AMX removal (97.9% removal at pH 5, contact time 30 min, q_max_ ≈40.28 mg/g)^8^. Although pomegranate-peel-based carbons have been explored, the number of studies evaluating their performance specifically for amoxicillin removal via pyrolysis-derived activated carbon from waste pomegranate peel is still limited^8–10^.

In this context, pyrolysis stands out as an efficient thermochemical method for converting agricultural residues into high-value products such as bio-oil and activated carbon. Pyrolysis-derived activated carbons display unique structural and surface properties that may differ significantly from those produced by conventional chemical activation, potentially offering improved adsorption performance depending on process parameters^11,12^.

This study’s novelty lies in the production of a high–surface area activated carbon from waste pomegranate peels via a simple KOH-assisted pyrolysis process and its application for amoxicillin removal from aqueous solutions^10^. Unlike previous studies that often employ nano-modified or composite adsorbents, the present work demonstrates that efficient AMX removal can be achieved without secondary modification. The prepared carbon exhibits a high surface area (1307 m² g⁻¹) and well-developed porosity, highlighting the advantages of simplicity, cost-effectiveness, and environmental sustainability. In this context, the study systematically investigates the effects of key operational parameters, evaluates equilibrium and kinetic behavior, and assesses the potential of this material as a scalable alternative for antibiotic removal.

## Materials and methods

### Materials

Waste pomegranate peels used as precursor material were obtained from local juice-processing facilities in Afyonkarahisar, Turkey. The activated carbon (AC) used in adsorption experiments was produced in our previous study by KOH chemical activation followed by pyrolysis^10^. Briefly: dried pomegranate peel powder was impregnated with KOH at a biomass: KOH mass ratio of 1:3, dried (105 °C, 24 h), carbonized at 800 °C for 1 h under N₂ flow (≈ 100 mL/min), washed with hot distilled water until neutral pH, dried (105 °C) and stored in airtight amber containers. Prior to use, the AC was ground and sieved (≤ 250 μm) to ensure homogenous particle size.

Amoxicillin trihydrate (≥ 98%), acetonitrile (HPLC grade, ≥ 99.9%), methanol (≥ 99%), ethanol (≥ 99%), NaOH, HCl, KH₂PO₄ and H₃PO₄ were purchased from Sigma-Aldrich (Turkey). Ultrapure water (18.2 MΩ·cm) was obtained from a Merck-Millipore Milli-Q system.

### Methods

#### Characterization of activated carbon

Surface morphology of the activated carbon was examined using scanning electron microscopy (SEM; Zeiss Sigma 300). Textural properties, including BET surface area, pore size distribution, and total pore volume, were determined by N₂ adsorption–desorption analysis using a Micromeritics Gemini VII analyzer. The point of zero charge (pHpzc) of the activated carbon was measured by the pH-drift method.

#### Determination of pHpzc value of activated carbon

The pHpzc value of activated carbon was determined through a series of steps. Initially, a 0.1 M NaCl solution was prepared. Subsequently, 50 mL aliquots of this solution were transferred into 250 mL volumetric flasks. The pH of each flask was adjusted to values of 2, 4, 6, 8, 10, and 12 using either 0.1 M HCl or 0.1 M NaOH solutions. Following pH adjustment, 0.050 g of activated carbon was added to each flask. The flasks were then placed on a shaker and agitated for a duration of 48 h at a constant temperature of 25 °C and a shaking speed of 150 rpm. A contact time of 48 h was selected based on literature and preliminary observations indicating stable pH values^13^. At the end of 48 h, each mixture was filtered through white band filter paper, and the pHf values of each sample were determined with a pH meter, and the pHf versus pHi curve was plotted^14^.

#### Batch adsorption experiments

Batch adsorption experiments were conducted in 250 mL capped flasks, utilizing 50 mL of the prepared Amoxicillin (AMX) solutions. Activated carbon was previously weighted and added to the solution, and then The flask was placed in a Thermomac DSL500 Orbital-Linear Shaker and a shaking speed of 180 rpm was selected to ensure sufficient mixing and to minimize external mass transfer limitations during the adsorption process. Samples taken after the experiments were filtered through a 0.22 μm injector tip membrane filter, and the AMX concentrations were measured in HPLC. In the adsorption experiments, the influence of solution pH (2,4,6,8,10), adsorbent dose (0.025, 0.5, 0.1 g/L), initial concentration (25,50,75 ppm), and temperature (25, 35, 45 °C) on the efficiency of AMX removal was systematically investigated. The pH of the solutions within the range of 2 to 10 was adjusted using 0.1 N HCl and 0.1 N NaOH solutions. The initial pH of the solutions was adjusted before the experiments, and since pH changes were generally minimal under the applied conditions, it was assumed that the pH value did not change during adsorption. All experiments were conducted under identical conditions to ensure consistency of the results. The amount of AMX adsorbed per unit mass of activated carbon at moment of t (*q*_*t*_) and at moment of equilibrium (*q*_*e*_) was calculated from the following equations:

1 $$\:{q}_{t}=\left(\frac{{C}_{0}-{C}_{t}}{w}\right)\times\:V$$

2 $$\:{q}_{e}=\left(\frac{{C}_{0}-{C}_{e}}{w}\right)\times\:V$$

Additionally, the percentage of AMX adsorbed was calculated using the following equation:

3 $$\:Remowing\:\left(\%\right)=\left(\frac{{C}_{0}-{C}_{e}}{{C}_{0}}\right)\times\:100\:$$

The initial concentration (*C*_*0*,_ mg/L), the equilibrium concentration (*Ce*, mg/L), the concentration at time of t (*C*_*t*_, mg/L), the volume of solution (*V*, L) and the mass of activated carbon (w, g)^15–17^.

#### Adsorption isotherms of the adsorption process

Adsorption isotherms provide essential insight into the interaction mechanisms between adsorbent and adsorbate molecules and are considered a fundamental tool for describing adsorption equilibria^18^. In this study, equilibrium adsorption experiments were conducted at three temperatures (25, 35 and 45 °C) to investigate the thermodynamic effects on the adsorption of amoxicillin (AMX). All isotherm experiments were performed under optimized conditions: initial concentration 50 mg/L, pH 6, and adsorbent dose 0.05 g.

Two widely used adsorption isotherm models—Langmuir and Freundlich—were applied to fit the equilibrium data, in accordance with recent literature^19,20^.

The Langmuir model assumes monolayer adsorption on a homogeneous surface^21^ and is expressed as:

4 $$\:\frac{{\mathrm{C}}_{\mathrm{e}}}{{\mathrm{q}}_{\mathrm{e}}}\mathrm{=}\frac{\mathrm{1}}{{\mathrm{q}}_{\mathrm{m}}{\mathrm{K}}_{\mathrm{L}}}\mathrm{+}\frac{{\mathrm{C}}_{\mathrm{e}}}{{\mathrm{q}}_{\mathrm{m}}}$$

where q_m_​ (mg g⁻¹) is the monolayer adsorption capacity and K_L_​ (L/mg) is the Langmuir constant. Adsorption favorability was evaluated using the dimensionless separation factor R_L_ ​:

5 $$\:{R}_{L}=\frac{1}{1+{K}_{L}{C}_{0}}$$

Values of 0 < R_L_ < 1 indicate favorable adsorption, R_L_ = 1 linear, R_L_ > 1 unfavorable, and R_L_ = 0 irreversible adsorption^22^.

The Freundlich model describes adsorption on heterogeneous surfaces^23^ and is expressed as:

6 $$\:{ln}{q}_{e}={ln}{K}_{F}+\frac{1}{n}\:{ln}{C}_{e}$$

where *K*_*F*_ (L/mg) represents adsorption capacity and 1/n describes adsorption intensity. Values of *n* > 1 indicate favorable adsorption^18^.

The equilibrium adsorption capacities (q_e_) at each temperature were calculated using Eq. (2), and the experimental data were fitted to both isotherm models to determine the adsorption mechanism and the effect of temperature on adsorption affinity^16^.

#### Kinetic modeling of the adsorption process

Kinetic analysis is essential for understanding the adsorption mechanism, identifying the rate-controlling steps, and determining how rapidly equilibrium is achieved. To evaluate the adsorption kinetics of AMX onto activated carbon, the pseudo-first-order (PFO) and pseudo-second-order (PSO) models were employed, which are among the most widely applied kinetic models in adsorption studies^24^.

The linear form of the pseudo-first-order model is expressed as:

7 $$\:{ln}\left({q}_{e}-{q}_{t}\right)={ln}{q}_{e}-{k}_{1}\:t$$

where k_1_ (1/min) is the PFO rate constant q_t_, (mg/g) is the adsorption capacity at time t, and q_e_​ (mg/g) is the equilibrium adsorption capacity.

The pseudo-second-order kinetic model is given by:

8 $$\:\frac{t}{{q}_{t}}=\frac{1}{{k}_{2}{q}_{e}^{2}}+\frac{1}{{q}_{e}}\:t$$

where k_2_ (g mg⁻¹ min⁻¹) is the PSO rate constant. The PSO model assumes that chemisorption may be the rate-controlling step and often provides a better fit for adsorption of organic pollutants onto carbonaceous materials^18^.

The kinetic parameters (k_1_, k_2_, q_e_) were obtained from the linear plots of Eqs. (7) and (8). The model providing the higher correlation coefficient (R²) was considered more appropriate for describing the adsorption kinetics of AMX on the prepared activated carbon.

#### Thermodynamic modeling of the adsorption process

Thermodynamic parameters, including the standard Gibbs free energy change (ΔG°), enthalpy change (ΔH°), and entropy change (ΔS°), provide critical insights into the feasibility, spontaneity, and energetic nature of adsorption processes. These parameters were evaluated by conducting equilibrium experiments at different temperatures and applying classical thermodynamic relationships^25^.

The standard Gibbs free energy change (ΔG°) for the adsorption of AMX onto the prepared activated carbon was calculated using the following equation:

9 $$\:\Delta G^{^\circ } = - RTInK_{e}$$

In this equation, K_e_ is the equilibrium constant, R is the gas constant (8.314 J/mol K), and T is the absolute temperature (K).

The equilibrium constant was calculated as:

10 $$\:{K}_{e}\mathrm{=}\frac{{q}_{e}}{{C}_{e}}$$

where q_e​_ (mg g^− 1^) is the equilibrium adsorption capacity, and C_e​_ (mg/L) is the equilibrium AMX concentration in solution.

The relationship between ΔG°, ΔH°, and ΔS° is represented by the fundamental thermodynamic equation:

11 $$\:\Delta G^{^\circ } = \Delta H^{^\circ } - {\mathrm{T}}\Delta S^{^\circ }$$

Combining Eqs. (9) and (11) leads to a van’t Hoff relationship, which allows ΔH° and ΔS° to be determined from the slope and intercept of the plot of In K_e​_ versus 1/T:

12 $$\:\mathrm{ln}{K}_{e}\mathrm{=-}\frac{{\varDelta\:G}^{^\circ\:}}{\mathrm{RT}}\mathrm{\:=\:}\frac{{\varDelta\:S}^{^\circ\:}}{\mathrm{R}}\mathrm{-}\frac{{\varDelta\:H}^{^\circ\:}}{\mathrm{RT}}\:$$

A positive value of ΔH° indicates an endothermic adsorption process, whereas the sign and magnitude of ΔS° reflect changes in randomness at the solid–solution interface. The spontaneity of AMX adsorption is confirmed by negative ΔG° values, with more negative values corresponding to stronger driving forces for adsorption^26,27^.

#### Analytical method and validation

Amoxicillin (AMX) concentrations in aqueous solutions were determined using a validated HPLC method. Chromatographic separation was performed on an Agilent ZORBAX Extend-C18 column (250 mm × 4.6 mm, 5 μm) at 25 °C. The mobile phase consisted of 10 mM KH₂PO₄ buffer (pH 2.0) and acetonitrile (92:8, v/v) and was applied isocratically at 1.0 mL/min. Detection was performed at 232 nm, and the AMX retention time was approximately 5.35 min.

The analytical method was validated according to ICH Q2(R1) guidelines. Linearity was assessed using standard AMX solutions in the range of 1–50 µg/mL, and calibration curves were obtained by plotting peak area against concentration. The sensitivity of the method (LOD and LOQ) was calculated from the calibration curve parameters. Precision, accuracy, and system suitability tests confirmed that the method is reliable for measuring AMX in adsorption experiments.

## Results and discussion

### Characterization of activated carbon

The activated carbon used in this study was produced from waste pomegranate peels through KOH-assisted chemical activation followed by pyrolysis, as detailed in our previous work^10^. Among the materials synthesized under different activation conditions, the sample obtained at 800 °C with a 3:1 KOH/biochar impregnation ratio exhibited the highest textural quality, with a BET surface area of 1307.0 m^2^/g as presented in Table 4 of the previous study^10^. Owing to its superior porosity and surface characteristics, this high–surface area activated carbon was selected for use in the AMX adsorption experiments in the present work. The selection of the optimal sample was based on its overall adsorption performance, reflecting the combined effects of surface area, pore structure, and surface functional groups.

**Fig. 1:**
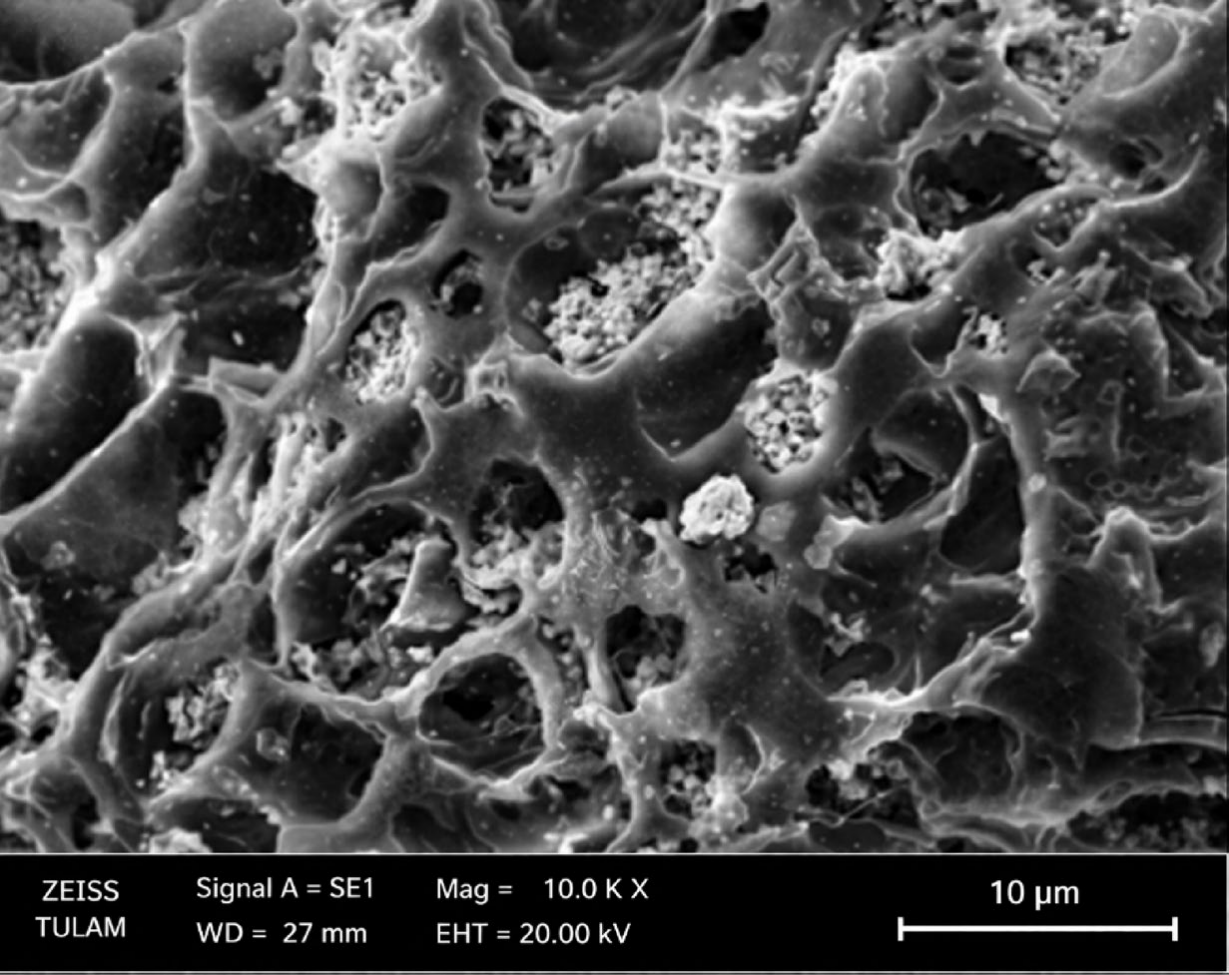
SEM image of activated carbon.

The SEM images (Fig. 1) show a well-developed porous structure consisting of interconnected micro- and mesopores typical of KOH-activated carbons. The activation process generated irregular pore walls and widened channels, providing abundant accessible adsorption sites for amoxicillin molecules.

Overall, the activated carbon produced from pomegranate peel exhibits the necessary structural and surface properties—high surface area, hierarchical porosity, and extensive pore accessibility—supporting its application as an efficient and sustainable adsorbent for antibiotic removal.

### Validation results of the analytical method

Standard solutions in the concentration range (1–50 µg/mL, *n* = 6) were prepared from the stock standard solution (500 µg/mL) by dilution with ultrapure water. Standard solutions were injected into HPLC system and peak areas and retention times of AMX were recorded. The same procedure was repeated for three consecutive days; the average peak area was calculated for each concentration level. A calibration graph was created, plotting peak area values against concentration. The regression equation, slope, and intercept were computed using the least squares method of linear regression. The linearity of the method was evaluated by the absolute mean recovery, RSD and R^2^ of the obtained calibration curve. The data of the validation studies are presented in Table 1.

**Table 1:** Validation results of the HPLC method.

Parameter	Value
Concentration range for linearity (µg mL^− 1^, *n* = 6)	1–50
Regression equation (y = mx+n)	$\:y=21.201\times\:-6.2533$
Coefficient of determination (R^2^)	0.9994
Retention time of FPR (min.)	4.35
Detection limit (LOD)/Quantification limit (LOQ) (µg mL^− 1^)	1.10/3.50
Recovery % [*n* = 3]	98.94–100.75
Accuracy R.S.D. % [*n* = 3]	0.223
Precision R.S.D. % [*n* = 3]	0.124

It was observed that the developed HPLC method met all the requirements of the validation process according to ICH guidelines and was accurate, linear, sensitive, precise and robust.

### Determination of zero adsorbent charge point (pHpzc)

**Fig. 2:**
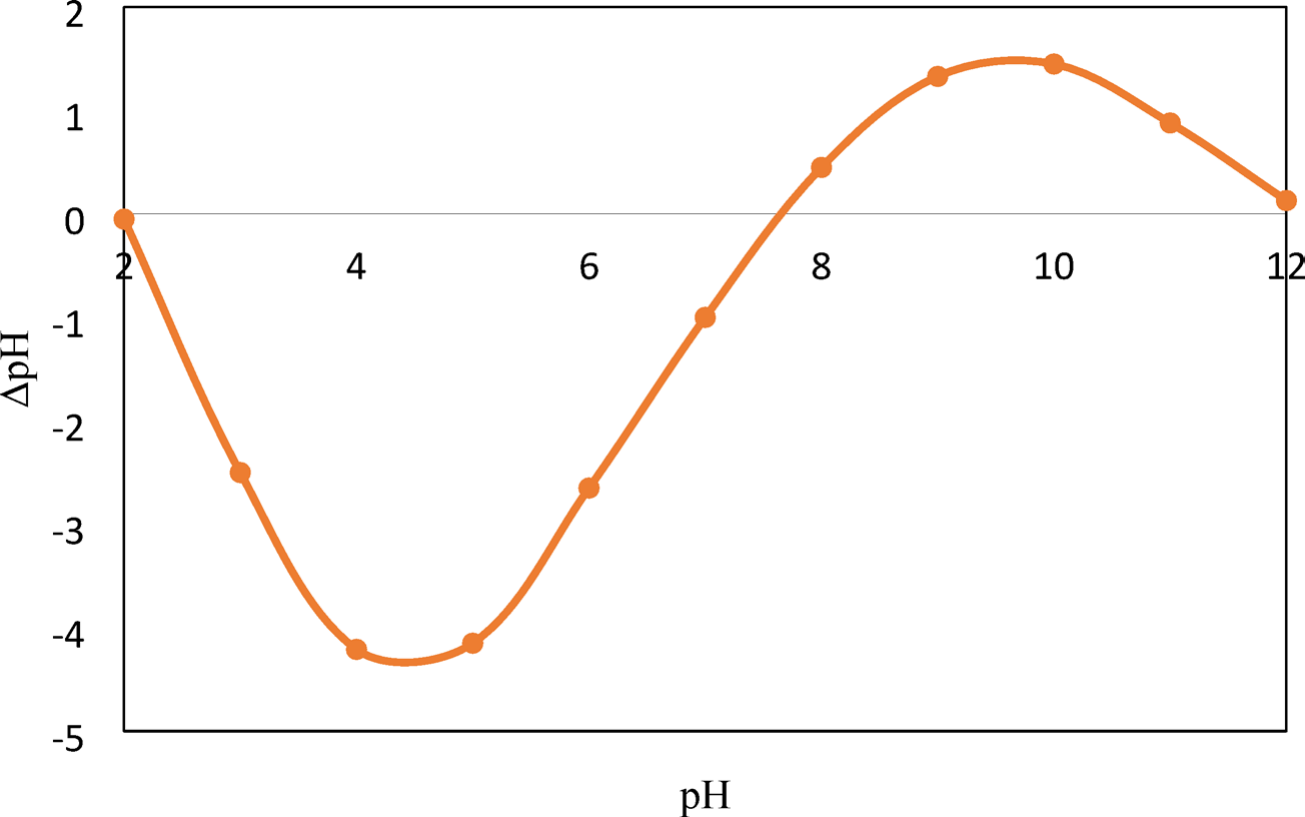
pH zero charge point of activated carbon (pHpzc).

The point of zero charge (pHpzc) of the pomegranate-peel-derived activated carbon was determined using the pH-drift method. Initial pH values (2–12) were adjusted in 0.1 M NaCl solutions, and equilibrium pH values were recorded after 48 h of contact. The intersection of pHf and pHi revealed a pHpzc value of 7.7 (Fig. 2).

This result indicates that the carbon surface is positively charged at pH < 7.7 and negatively charged at pH > 7.7. Considering the amphoteric behavior of amoxicillin, which predominantly exists in cationic/neutral forms under acidic conditions, the adsorption process cannot be explained solely by electrostatic interactions. This finding is consistent with the high AMX removal efficiencies (≈ 90–94%) observed at acidic pH in this study.

### The effect of pH on adsorption performance

**Fig. 3:**
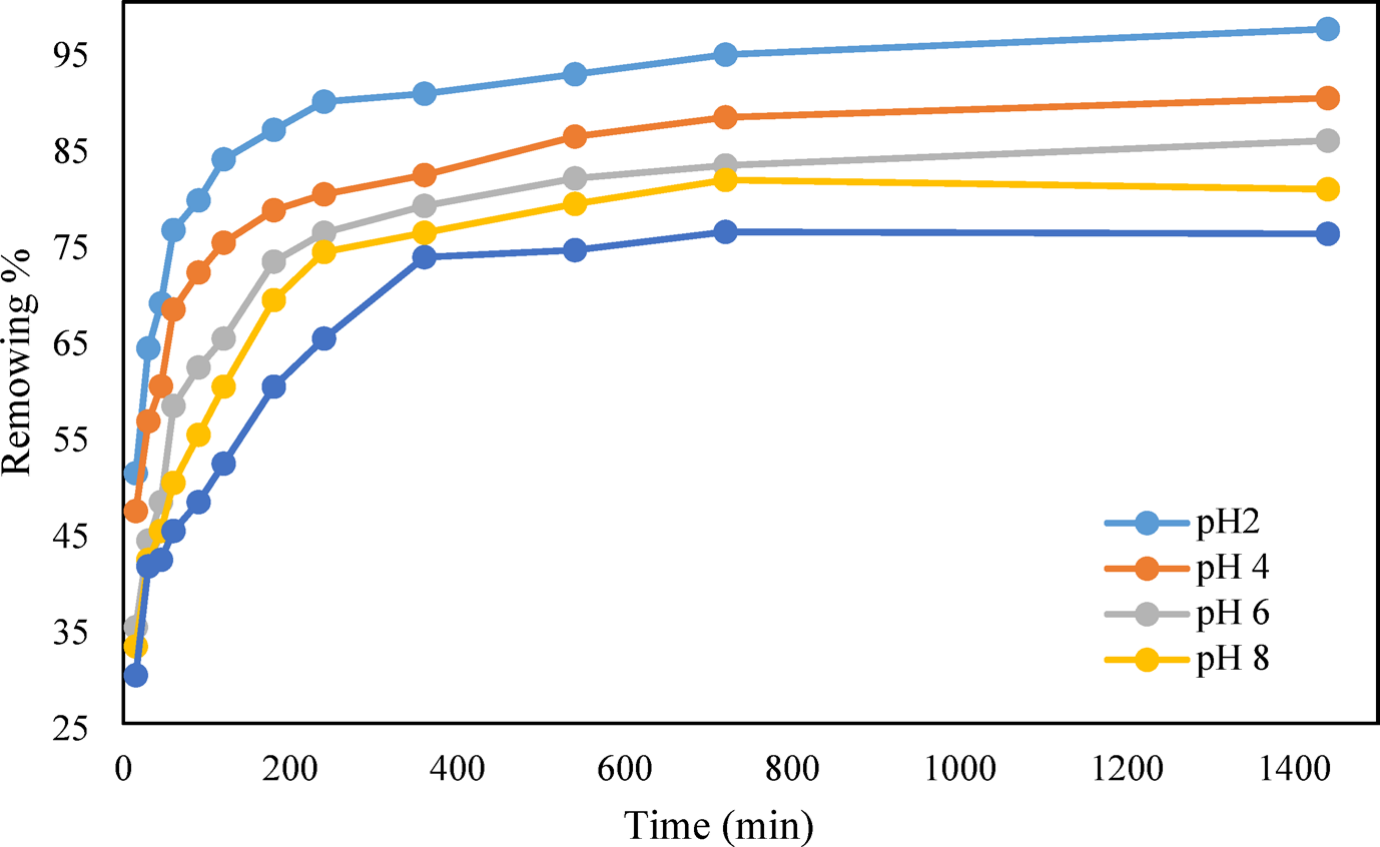
The effect of pH on AMX Adsorption.

The adsorption of amoxicillin (AMX) onto the pomegranate peel–derived activated carbon was strongly dependent on the solution pH (Fig. 3). The removal efficiency was highest under acidic conditions, reaching approximately 97% at pH 2 and 90% at pH 4. As the pH increased toward neutrality, adsorption slightly decreased (**~** 87% at pH 6**)**, and the lowest removal occurred under alkaline conditions (**~** 79% at pH 10).

This behavior is governed by the surface charge of the activated carbon and the pH-dependent speciation of amoxicillin (AMX). The pHpzc value (7.7) indicates that the adsorbent surface is positively charged at pH < 7.7 and negatively charged at pH > 7.7. However, the adsorption mechanism cannot be explained solely by electrostatic interactions. At strongly acidic conditions (pH 2–4), AMX predominantly exists in its cationic form. Electrostatic repulsion may occur between the positively charged surface and AMX molecules. However, the high removal efficiency observed in this study suggests that non-electrostatic interactions. At near-neutral pH, where AMX exists mainly in its zwitterionic form, both attractive and repulsive interactions may occur, resulting in slightly reduced but still significant adsorption. At alkaline pH, AMX is predominantly in its anionic form, and the negatively charged adsorbent surface leads to electrostatic repulsion, which contributes to the observed decrease in adsorption efficiency. Therefore, the adsorption behavior is governed by a complex interplay between electrostatic interactions and other surface-controlled mechanisms, rather than electrostatic attraction alone.^6,28^. Overall, the results confirm that acidic conditions (pH 2–4) provide the most efficient AMX removal.

Although the highest removal efficiency was obtained under acidic conditions, this may limit direct applicability in real wastewater treatment systems, where pH is typically near neutral. However, acceptable removal efficiencies can still be achieved at near-neutral pH values. In addition, process optimization strategies such as increasing adsorbent dosage or contact time may improve performance under practical conditions.

### The effect of adsorbent dose on adsorption performance

**Fig. 4:**
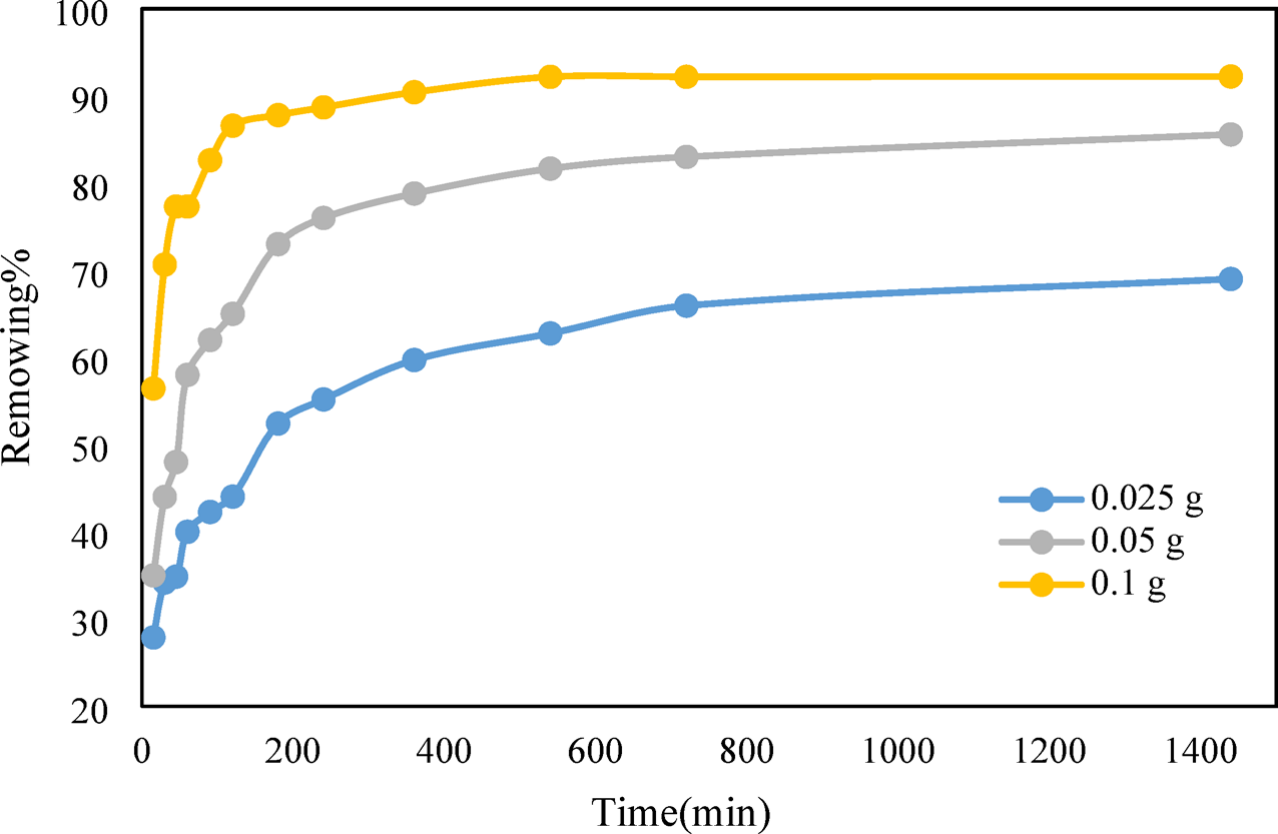
The effect of adsorbent dose on adsorption performance.

The adsorbent dose had a substantial influence on the removal efficiency of AMX, as shown in Fig. 4. Increasing the activated carbon dosage from 0.025 g to 0.10 g resulted in a remarkable enhancement in adsorption performance. At the lowest dose (0.025 g), the maximum AMX removal reached only 69%, whereas increasing the dose to 0.05 g improved the removal efficiency to 86%. The highest performance was observed at 0.10 g, where the removal exceeded 92%, indicating the presence of abundant active sites and improved pollutant–adsorbent contact.

The improvement in removal with increasing dose can be attributed to the higher availability of surface functional groups and adsorption sites, which accelerates the uptake of AMX molecules and shortens the time required to approach equilibrium. Similar trends have been reported for AMX adsorption onto different types of activated carbons and carbonaceous materials, where increasing the adsorbent mass enhances adsorption efficiency due to a larger number of accessible adsorption sites^29,30^.

However, although the removal efficiency increased with adsorbent dose, the adsorption capacity per unit mass (qe) typically decreases at higher doses due to the unsaturated occupation of available sites and particle aggregation, a commonly reported phenomenon in adsorption systems^31^. These observations indicate that while larger adsorbent doses improve total AMX removal, there is an optimal dose beyond which further increases may not significantly enhance adsorption efficiency.

Therefore, 0.05 g was selected as the optimal adsorbent dose as it provides a reasonable compromise between high removal efficiency and efficient adsorbent utilization, which is important for practical applications.

### The effect of initial concentration on adsorption performance

**Fig. 5:**
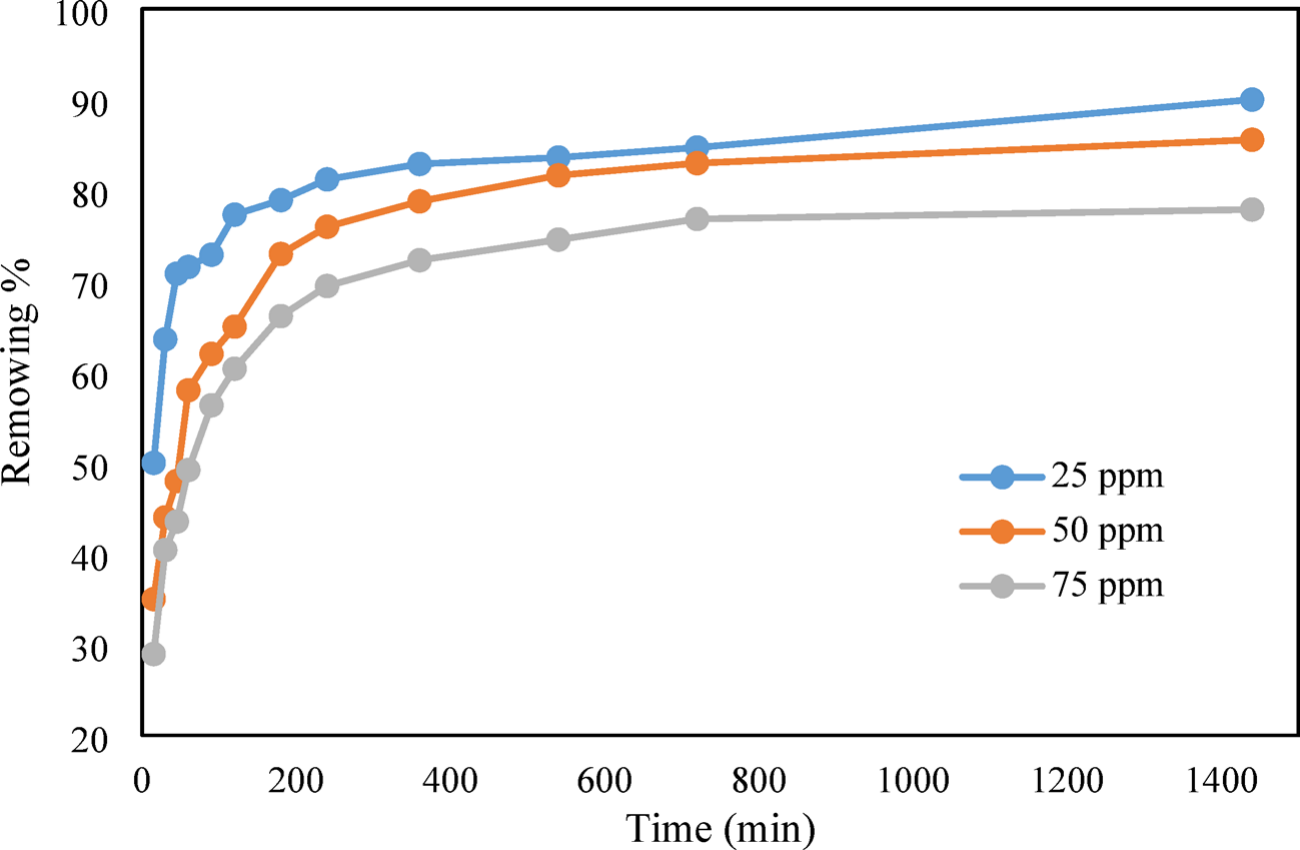
The effect of initial concentration on adsorption performance.

The initial concentration of AMX had a noticeable impact on the adsorption efficiency (Fig. 5). As the concentration increased from 25 to 75 ppm, the removal percentage decreased. The highest removal (90%) was obtained at 25 ppm, while 86% and 78% removals were recorded at 50 ppm and 75 ppm, respectively. At low concentrations (25 ppm), the available active sites on the activated carbon surface were sufficient to capture most AMX molecules, resulting in high removal. However, at higher concentrations, the ratio of solute molecules to available adsorption sites increased, causing partial site saturation and reduced removal efficiency.

This behavior aligns with previous studies. Touijer et al. ^32^ reported a similar trend for biomass-derived activated carbon, showing that AMX removal decreased at higher initial concentrations due to limited active site availability. Likewise, a study using mineral-activated bituminous carbon confirmed that increased AMX concentration reduced removal efficiency as adsorption sites approached saturation^33^. Recent work on chitosan-derived porous carbon also demonstrated a comparable decline in AMX adsorption efficiency with increasing initial concentration, attributing this to surface saturation effects^6^.

Overall, these findings indicate that lower initial AMX concentrations favor higher removal efficiency due to an improved balance between solute molecules and available adsorption sites.

### The effect of temperature on adsorption performance

**Fig. 6:**
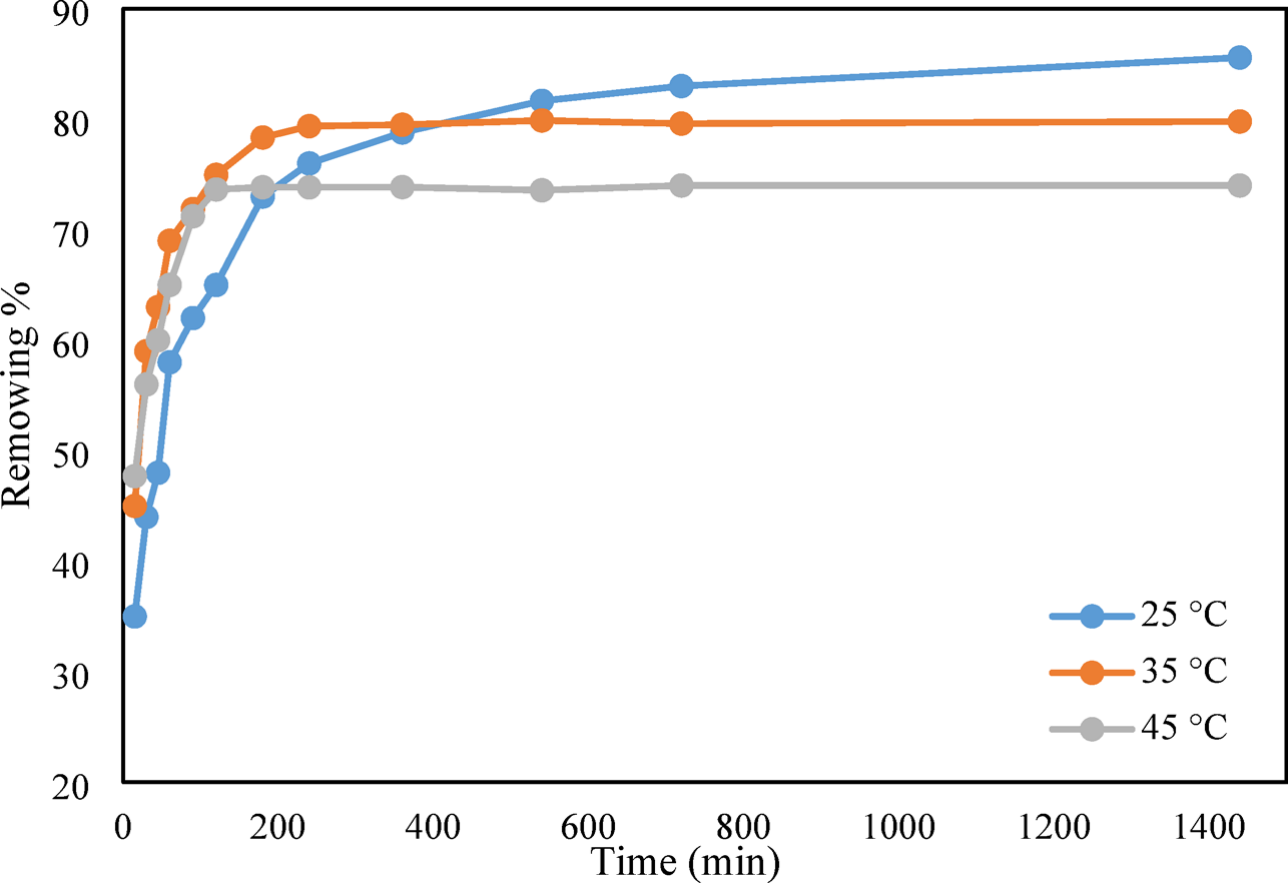
The effect of temperature on the adsorption performance.

Temperature had a distinct effect on AMX adsorption (Fig. 6). The highest removal efficiency was obtained at 25 °C (86%), while lower efficiencies were recorded at 35 °C (80%) and 45 °C (74%). The decline in removal with increasing temperature indicates an exothermic adsorption process, as higher thermal motion weakens the interactions between AMX molecules and the activated carbon surface^32^.

Studies investigating antibiotic adsorption onto various carbon-based materials also report that elevated temperatures reduce adsorption efficiency due to decreased affinity between adsorbate molecules and active surface sites^34^. Similar exothermic behavior has been noted for AMX removal using porous carbon structures, further supporting that lower temperatures enhance adsorption performance^6^. Therefore, 25 °C was selected as the optimal temperature, as higher temperatures reduced adsorption efficiency due to the exothermic nature of the process.

### Adsorption kinetics

**Fig. 7:**
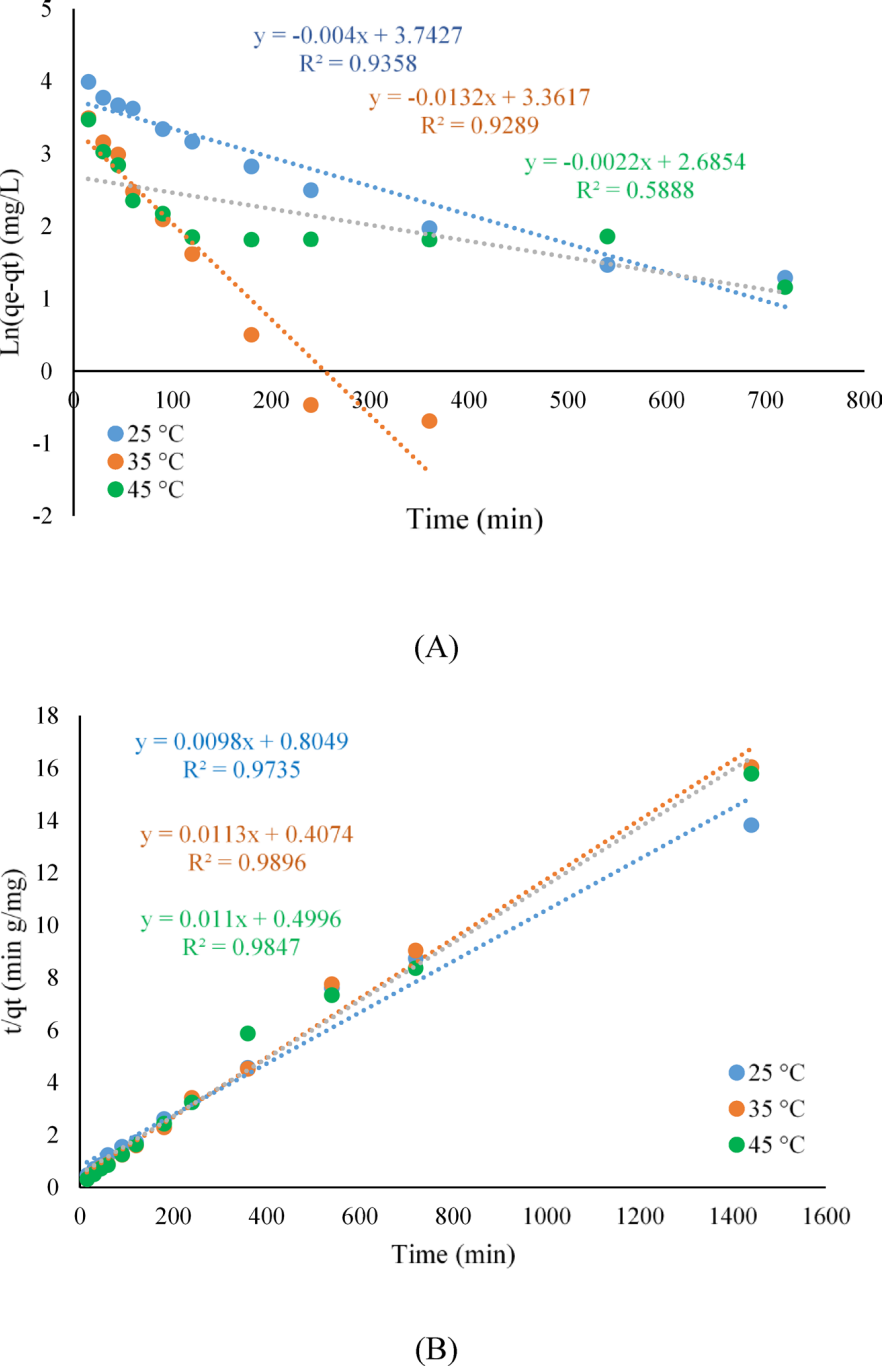
(**A**) Pseudo-first-order and (**B**) Pseudo-second-order models (Contact time: 24 h, pH: 6, adsorbent dose: 0.05 g, initial concentration: 50 mg/L).

The adsorption kinetics of amoxicillin (AMX) onto pomegranate peel–derived activated carbon were analyzed using pseudo-first-order (PFO) and pseudo-second-order (PSO) models at 25, 35, and 45 °C (Fig. 7; Table 2). The adsorption process exhibited a typical two-stage behavior, characterized by a rapid initial uptake followed by a slower approach to equilibrium. This pattern is commonly attributed to the abundance of readily available surface active sites at the beginning and subsequent intraparticle diffusion limitations as adsorption progresses.

A clear distinction between the two kinetic models was observed. The PFO model showed relatively poor agreement with the experimental data, particularly at higher temperatures (R² = 0.5888 at 45 °C), and significantly underestimated the equilibrium adsorption capacity (qe, cal < < qe, exp). This deviation indicates that the adsorption process cannot be adequately described by a simple first-order diffusion-controlled mechanism.

In contrast, the PSO model provided an excellent fit across all temperatures, with high correlation coefficients (R² = 0.9735–0.9896) and close agreement between calculated and experimental qe values. Furthermore, significantly lower RMSE (0.98–1.21) and χ² (1.87–2.45) values confirmed the superior predictive capability of the PSO model. These findings suggest that the overall adsorption rate is governed by surface interactions rather than solely by external mass transfer.

Although the PSO model is often associated with chemisorption, it should not be interpreted exclusively as evidence of chemical bonding. Instead, it may reflect complex adsorption mechanisms involving multiple interactions, such as π–π interactions, hydrogen bonding, and electrostatic attraction, as well as intraparticle diffusion effects. Similar kinetic behavior has been widely reported for AMX adsorption onto carbon-based and biomass-derived adsorbents, where PSO consistently provides the best fit to experimental data^32,33^.

Additionally, the slight variation of the PSO rate constant (k₂) with temperature suggests that increasing temperature does not enhance adsorption kinetics, supporting the exothermic nature of the process. This observation is consistent with previous studies reporting decreased antibiotic adsorption rates at elevated temperatures due to weakened adsorbate–adsorbent interactions^6,34^.

Overall, the results demonstrate that AMX adsorption onto the prepared activated carbon is best described by the pseudo-second-order kinetic model, indicating that surface-controlled mechanisms dominate the rate-limiting step, with possible contributions from diffusion processes. This suggests that surface-controlled interactions are the dominant rate-limiting step, while intra-particle diffusion may contribute at later stages of the adsorption process.

**Table 2:** Parameters of kinetic model for adsorption process.

Type of kinetic model	Parameters of the kinetic model	Temperature		
		25 °C	35 °C	45 °C
Pseudo first order	k_1_ (1/min)	0.004	0.0132	0.0022
	Calculated q_e_ (mg/g)	42.21	28.84	14.66
	Experimental q_e_ (mg/g)	86	80	80
	R^2^	0.9358	0.9289	0.5888
	RMSE	2.84	3.12	5.76
	χ²	8.12	9.45	18.32
Pseudo second order	k_2_ (g/mg min)	0.00012	0.00031	0.00024
	Calculated q_e_ (mg/g)	100.04	88.5	90.91
	Experimental q_e_ (mg/g)	86	80	80
	R^2^	0.9735	0.9896	0.9847
	RMSE	1.21	0.98	1.05
	χ²	2.45	1.87	2.02

### Adsorption isotherm

**Fig. 8:**
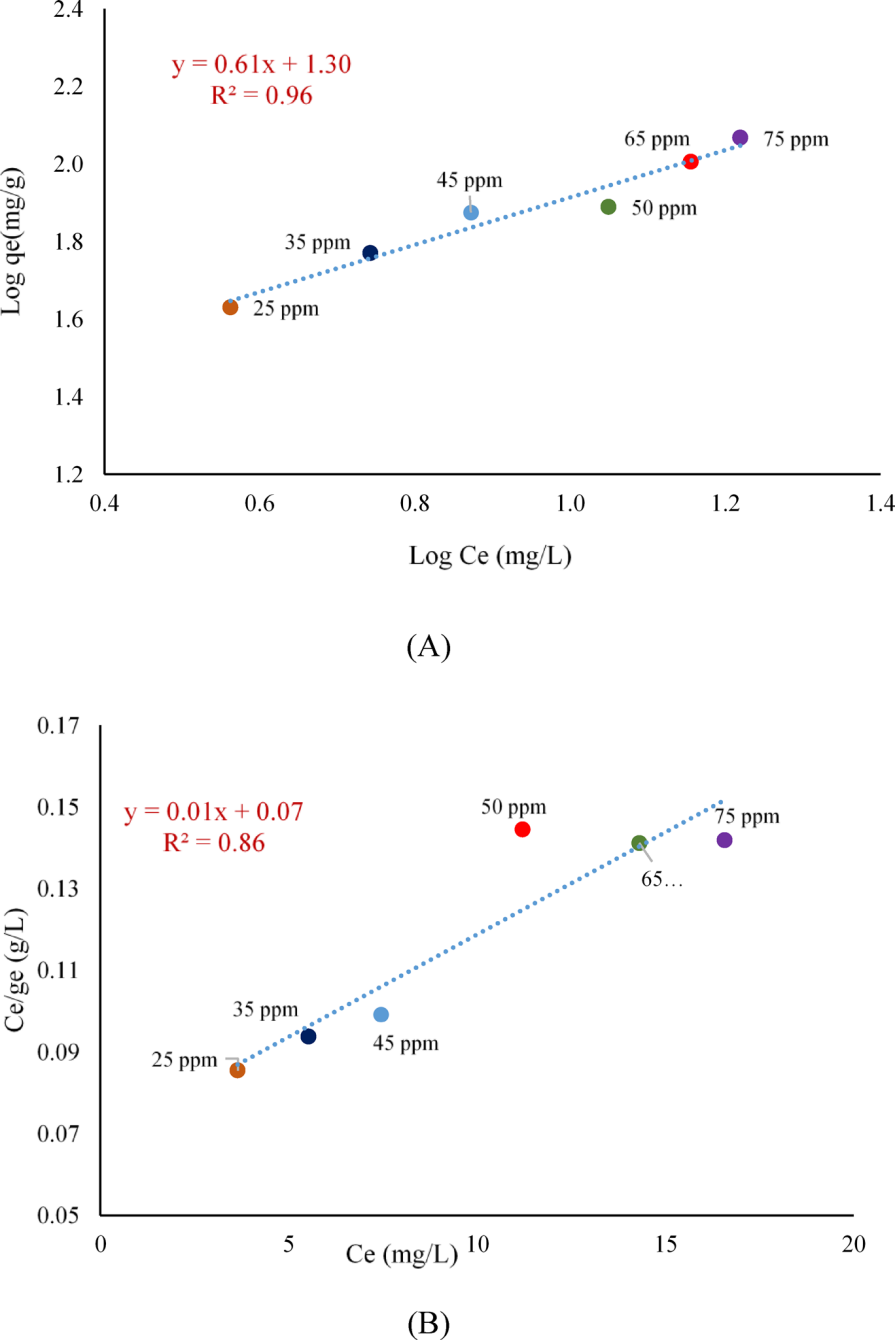
(**A**) Freundlich and (**B**) Langmuir isotherms (tempature: 25 °C, pH: 6, adsorbent dose: 0.05 g, contact time: 24 h).

The equilibrium adsorption behavior of amoxicillin (AMX) onto pomegranate peel–derived activated carbon was evaluated using Langmuir and Freundlich isotherm models based on equilibrium data (Ce and qe). The corresponding isotherm plots are presented in Fig. 8, and the model parameters are summarized in Table 3.

Both isotherm models described the experimental data; however, the Freundlich model exhibited a higher correlation coefficient (R^2^ = 0.96) than the Langmuir model (R^2^ = 0.86), indicating a slightly better fit. This suggests that adsorption occurs on a heterogeneous surface with a non-uniform distribution of active sites, which is typical of biomass-derived activated carbons^17,35^.

The Freundlich constants further support favorable adsorption, with n (1.639) > 1, indicating a strong interaction between AMX molecules and the activated carbon surface. It is well established that n values between 1 and 10 correspond to favorable adsorption conditions^18^. The K_F_ value (19.95 mg g⁻¹ (L/mg)¹⁄ⁿ) also reflects a relatively high adsorption capacity, consistent with previous studies on antibiotic removal using lignocellulosic adsorbents^36^.

The Langmuir model yielded a maximum monolayer adsorption capacity (q_max_) of 100 mg/g, suggesting a high theoretical adsorption capacity of the adsorbent. The Langmuir constant (K_L_ = 0.143 L/mg) indicates a moderate affinity between AMX and the adsorbent surface. According to Langmuir theory, this model assumes monolayer adsorption onto a surface with finite and identical adsorption sites^21^.

The dimensionless separation factor (R_L_), calculated from the Langmuir constant, ranged from 0.085 to 0.218 over the studied concentration range. Since all R_L_ values lie within 0 < R_L_ < 1, the adsorption process is considered favorable^37^. Lower R_L_ values at higher initial concentrations indicate stronger adsorption affinity under these conditions.

Overall, the results indicate that although both models can describe adsorption behavior, the Freundlich model provides a slightly better fit, suggesting that AMX adsorption occurs on a heterogeneous surface with possible multilayer formation. However, the relatively high qmax value obtained from the Langmuir model also indicates that monolayer adsorption plays a significant role in the adsorption mechanism. Similar dual behavior has been reported in previous studies investigating antibiotic adsorption onto activated carbons^38^.

In comparison with previously reported pomegranate peel-derived adsorbents, many studies involve additional modification steps such as nanoparticle incorporation or chemical functionalization. For instance, nZVI-modified pomegranate peel carbon exhibits adsorption capacities around 40 mg/g ^8^. In contrast, the activated carbon developed in this study achieves a higher adsorption capacity (q_max_ = 100 mg/g) without requiring secondary modification. This highlights the effectiveness, simplicity, and potential scalability of the proposed material.

**Table 3:** Parameters of isotherm models.

Model	Parameter	Value
Freundlich	K_F_ (mg/g^− 1^) (L/mg)^1/n^	19.95
	n	1.639
	R^2^	0.96
Langmuir	q_max_ (mg/g)	100
	K_L_ (L/mg)	0.143
	R_L_	0.218-0.166-0.134-0.097-0.085
	R^2^	0.86

### Thermodynamics of adsorption

The thermodynamic parameters derived from the van’t Hoff plot (Table 4) provide important insights into the nature of AMX adsorption onto the pomegranate peel–derived activated carbon. The Gibbs free energy values (ΔG° = −6.13, − 5.64, and − 5.16 kJ/mol at 293, 308, and 323 K, respectively) are negative at all temperatures, confirming that the adsorption process is spontaneous. However, the decrease in the magnitude of ΔG° with increasing temperature indicates reduced spontaneity at elevated temperatures, suggesting weaker adsorbate-adsorbent interactions^39,40^.

The enthalpy change (ΔH° = -15.59 kJ/mol) is negative, indicating an exothermic adsorption process. The relatively low magnitude of ΔH° (< 40 kJ/mol) suggests that the adsorption is mainly governed by physisorption mechanisms, such as electrostatic interactions, hydrogen bonding, and π–π interactions, rather than strong chemisorption^41,42^.

The negative entropy change (ΔS° = −32.29 J /mol K) indicates a decrease in randomness at the solid–liquid interface during adsorption. This implies that AMX molecules become more ordered upon adsorption onto the activated carbon surface, likely due to restricted mobility and structured arrangement within the porous network. Comparable entropy behavior has been observed in similar adsorption systems, where decreased interfacial randomness is associated with the formation of stable adsorbate–adsorbent interactions^16,43^.

Overall, the combination of negative ΔG°, ΔH°, and ΔS° values confirms that the adsorption process is spontaneous, exothermic, and accompanied by increased structural ordering at the interface. These findings are in good agreement with previously reported thermodynamic behaviors for antibiotic adsorption onto carbonaceous materials, further supporting the proposed adsorption mechanism.

**Table 4:** Thermodynamic parameters of the adsorption process.

Thermodynamic parameters	ΔG^o^ (kJ/mol)			ΔH^o^ (kJ/mol)	ΔS^o^ (J mol/K)
Temperature (K)	293	308	323	− 15.59	− 32.29
	− 6.13	− 5.64	− 5.16		

**Fig. 9:**
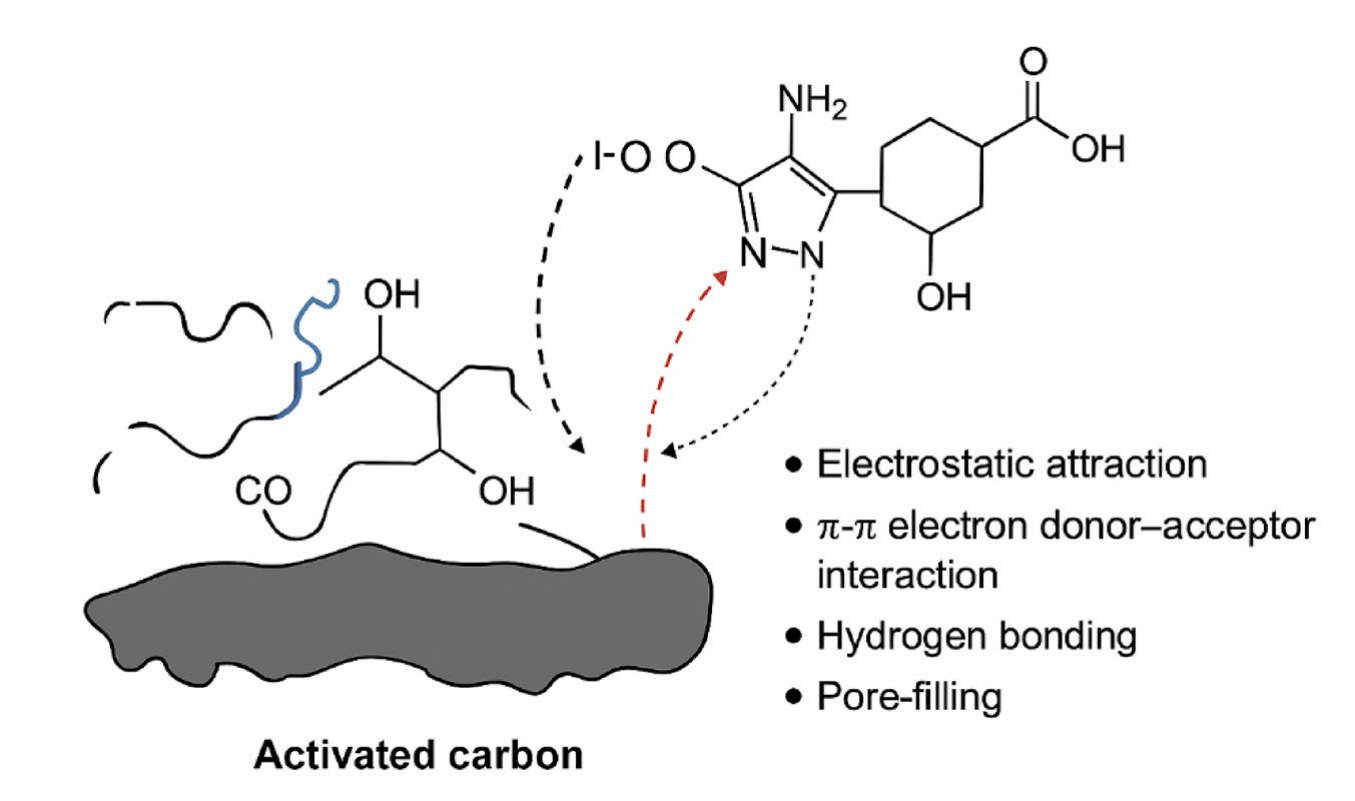
Adsorption mechanism of AMX onto pomegranate-peel-derived activated carbon.

The adsorption of AMX onto the pomegranate-peel-derived activated carbon can be described as a multi-mechanistic process predominantly governed by physisorption. The main interactions are electrostatic attraction, π–π donor–acceptor interactions, hydrogen bonding, and pore filling (Fig. 9).

The pHpzc value (7.7) indicates that the adsorbent surface is positively charged at pH < 7.7, favoring the uptake of protonated AMX species under acidic conditions and accounting for the enhanced removal efficiency observed at pH 2–4. Moreover, the aromatic structure of AMX facilitates π–π interactions with the graphitic domains of the carbon matrix, while oxygen- and nitrogen-containing surface functional groups (–OH, –COOH, –NH₂) may contribute to hydrogen bonding.

The high BET surface area (1307 m²/g) and well-developed micro–mesoporous structure further promote adsorption via pore filling. Although the pseudo-second-order (PSO) model provides the best fit to the kinetic data, it should not be taken as definitive evidence of chemisorption; rather, it reflects the dominance of surface-controlled interactions. Consistent with the revised thermodynamic findings (ΔH° < 40 kJ/mol), the overall adsorption mechanism is primarily physical, with possible minor contributions from stronger interactions.

These proposed mechanisms are consistent with the experimental findings obtained in this study. The strong adsorption under acidic conditions supports the role of non-electrostatic interactions, while the pseudo-second-order kinetic model indicates the importance of surface-controlled processes. In addition, the thermodynamic results (ΔH° < 40 kJ/mol) suggest that physisorption dominates, supporting the interactions illustrated in Fig. 9.

## Conclusion

The present study demonstrates that activated carbon derived from waste pomegranate peels via a simple KOH-assisted pyrolysis process is an effective adsorbent for the removal of AMX from aqueous solutions. The material exhibited a high surface area (1307 m²/g) and well-developed porosity, enabling efficient adsorption performance, with a maximum removal efficiency of 97% achieved under optimal conditions (pH 2, 0.05 g, 25 °C, 50 mg/L), while maintaining 87% removal at near-neutral pH.

Adsorption performance was strongly influenced by operational parameters. Increasing adsorbent dose enhanced removal efficiency due to the availability of additional active sites, whereas higher initial concentrations led to reduced removal due to site saturation. The decrease in adsorption efficiency with increasing temperature confirmed the exothermic nature of the process.

Kinetic analysis indicated that the pseudo-second-order model best describes the adsorption process, reflecting the dominance of surface-controlled interactions. Equilibrium data suggested heterogeneous adsorption behavior, with the Freundlich model providing a slightly better fit, although Langmuir analysis also indicated a high monolayer adsorption capacity (q_max_ = 100 mg/g).

Thermodynamic evaluation revealed that the adsorption is spontaneous (ΔG° < 0) and exothermic (ΔH° = −15.59 kJ/mol), with decreased randomness at the solid–liquid interface (ΔS° < 0). The relatively low enthalpy value confirms that the adsorption mechanism is predominantly governed by physisorption, involving electrostatic attraction, π–π interactions, hydrogen bonding, and pore filling.

Overall, the results show that this low-cost and sustainable adsorbent is a promising option for antibiotic removal from water. The developed material offers strong potential for practical application in wastewater treatment. Spent adsorbent can be regenerated or safely disposed of in accordance with environmental regulations. Future work should focus on regeneration, reusability, and continuous-flow system performance to evaluate its industrial applicability.

A limitation of this study is that experiments were conducted using synthetic solutions. The presence of competing species in real wastewater may affect adsorption performance; therefore, further studies are needed.

## Data Availability

Data will be made available on request.

## Declarations

### Author statement

This is to state that this work is original and is not submitted to any other journal/publication. All the authors have consented to submit the manuscript.
